# Controversial Link between Cannabis and Anticancer Treatments—Where Are We and Where Are We Going? A Systematic Review of the Literature

**DOI:** 10.3390/cancers14164057

**Published:** 2022-08-22

**Authors:** Bianca Hanganu, Diana Elena Lazar, Irina Smaranda Manoilescu, Veronica Mocanu, Doina Butcovan, Camelia Liana Buhas, Andreea Silvana Szalontay, Beatrice Gabriela Ioan

**Affiliations:** 1 Department of Forensic Medicine, Faculty of Medicine, Grigore T. Popa University of Medicine and Pharmacy, 700115 Iasi, Romania; 2 Department of Oncology, Municipal Hospital “St. Hierarch Dr. Luca”, 601048 Onesti, Romania; 3 Department of Morpho-Functional Sciences (Pathophysiology), “Grigore T. Popa” University of Medicine and Pharmacy, 16, Universitatii Street, 700115 Iasi, Romania; 4 Department of Morpho-Functional Sciences (Morphopathology), “Grigore T. Popa” University of Medicine and Pharmacy, 16, Universitatii Street, 700115 Iasi, Romania; 5 Department of Pathology, “Prof. George Georgescu” Institute of Cardiovascular Diseases, 50, Carol I Avenue, 700503 Iasi, Romania; 6 Department of Morphological Disciplines, Faculty of Medicine and Pharmacy, University of Oradea, 410087 Oradea, Romania; 7 Department of Psychiatry, Faculty of Medicine, Grigore T. Popa University of Medicine and Pharmacy, 700115 Iasi, Romania

**Keywords:** cannabidiol, cannabis, dronabinol, endocannabinoids, medical marijuana, nabiximols, nabilone, cancer

## Abstract

**Simple Summary:**

In the field of oncology, preclinical research has shown that cannabis and cannabinoids modulate signaling pathways involved in cell proliferation, migration, invasion, angiogenesis, programmed cell death, and metastasis. Based on these findings, as medical cannabis becomes legal in more and more countries, cancer patients and their families are increasingly interested in the potential benefits of herbal medicine as an element of complementary and alternative medicine in their treatment. Although its clinical efficacy has been demonstrated in preclinical studies, clinical trials with cancer patients are lacking. To draw clear conclusions, we await the results of further prospective and randomized studies on this clinically relevant topic.

**Abstract:**

Background and Objectives: Cannabinoids are currently used in cancer patients primarily for their pain-relieving and antiemetic properties. The aim of our review was to synthesize all available data of studies evaluating the therapeutic efficacy of cannabis in combination with oncological treatments in cancer patients and to explore ongoing studies with different goals and medical areas registered in the field of oncology worldwide. Materials and Methods: This study was performed in accordance with the PRISMA guidelines. A search using MEDLINE/PubMed database was performed between 1 January 2006 and 1 March 2022. Search terms included the following: cannabidiol, cannabis, CBD, dronabinol, endocannabinoids, medical marijuana, nabiximols, nabilone, THC, and cancer. All studies that examined the efficacy of cannabis administered during oncological treatments, regardless of cancer localization, subtype, and sample size, were considered eligible. Results: In three studies, cannabis was administered to patients with glioblastoma, and in two other studies, cannabis was used in combination with immunotherapy in various cancer subgroups. The results of the clinical trials in cancer patients are not sufficient to draw conclusions at this time. Interestingly, several other studies addressing the systemic effects of cannabinoids in cancer patients are currently listed in the U.S. National Library of Medicine’s registry on the ClinicalTrials.gov website. However, only one of the registered studies examined the efficacy of cannabinoids as a potential option for systemic cancer treatment. Conclusions: Although cannabis is touted to the public as a cancer cure, clinical trials need to clarify which combinations of chemotherapeutic agents with cannabinoids are useful for cancer patients.

## 1. Introduction

Cannabis sativa (also known as marijuana, bhang, ganja, or hemp) [1] originated in Central Asia, being a significant source of cannabinoids (CBs) and the most commonly used illicit drug in Western countries [2,3].

Over the period 2010–2017, 159 countries covering 97% of the world’s total population reported cannabis plant cultivation to the United Nations Office on Drugs and Crime [4].

Humans have been ingesting or inhaling the cannabis plant for approximately 4000 years [5], but it is increasingly being criticized as a medicine [6]. Cannabis has received little attention from the scientific community in recent years due to its status as a controlled substance [7]. Consequently, cannabis as a whole plant product has not been clinically studied for the treatment of malignancies.

The cannabis plant contains about 421 compounds, 61 of which are cannabinoids [8]. The female flowers of the plant have the highest concentration of cannabinoids [9]. When cannabis is burned, pyrolysis produces about 2000 chemicals [10]. Various chemical classes, including nitrogenous compounds, amino acids, hydrocarbons, sugars, terpenes, and simple fatty acids, act together to give cannabis its various pharmacological and toxicological effects [11].

The two main constituents of the Cannabis sativa plant, delta-9 tetrahydrocannabinol (delta-9 THC) and cannabidiol (CBD), have unique clinical and behavioral effects, including experiencing a “high” sensation and tranquility/relaxation [12]. CBD has sedative properties [12] and can reduce the acute psychotic symptoms caused by delta-9 THC [13]. The main psychoactive ingredient of the Cannabis sativa plant, delta-9 THC, is thought to be responsible for the plant’s cognitive effects, psychiatric symptoms, and anxiety, as well as for the addictive potential of smoked cannabis [14,15]. The amount of delta-9 THC found in different countries, cannabis products, and genetic variants varies widely. These chemicals may have opposing effects on regional brain functions, which could explain their opposing symptomatic and behavioral effects, as well as the ability of CBD to block the psychotogenic effects of delta-9 THC [12,13,14,15,16]. Medical cannabis has many potential benefits but also a number of drawbacks.

Endocannabinoids, sometimes referred to as endogenous cannabinoids, are lipid metabolites that are crucial for intercellular communication in both juxtacrine and paracrine ways [14]. Endocannabinoids control a variety of physiological and pathological states (such as the regulation of appetite, analgesia, cancer, and addiction) by functioning as synaptic circuit breakers [15].

There are cannabinoid receptors at the supraspinal, spinal, and peripheral levels. By activating the cannabinoid receptor subtypes CB1 and CB2, cannabinoids reduce behavioral responses to noxious stimuli and nociceptive processing [15]. CB1 receptors are mainly located in the presynaptic neurons of the central nervous system and are responsible for the immediate psychological and cardiovascular effects of cannabis. CB2 receptors are mainly found in the periphery of the brain and regulate immunological function, as well as the inflammatory response [17,18,19,20]. Cannabinoids induce programmed cell death by complexing with the CB1 receptor [21]. This interaction also leads to increased inhibition of vascular endothelial growth factor [22], inhibiting angiogenesis and reducing tumor viability [23]. In vitro studies show that cannabinoids inhibit matrix mettaloproteinase-2, which allows cancer cells to invade and metastasize [22].

*The effects of cannabis on cancer*

*Cannabis sativa* plant extracts have always garnered a significant amount of interest in medicine, but now more than ever, with the burden of cancer on the population as a whole rising and the advent of new potential classes of medications, they provide a source of hope. Currently, the use of cannabis for medical purposes is authorized in 44 nations and territories worldwide [23,24].

Munson et al. provided the first evidence of the anticancer properties of cannabis in 1975 [25]. They showed that 9-tetrahydrocannabinol reduced the growth of lung adenocarcinoma cells in an in vitro cell line and in a mouse model after oral administration. On the other hand, cannabis use has been associated with head and neck cancer [26,27], lung cancer [28,29,30], laryngeal cancer [31], prostate cancer [32], testicular cancer [33,34,35,36], cervical cancer [37], brain cancer [38], and urothelial carcinoma [39,40,41]. Several pediatric cancers, including childhood neuroblastoma [42], rhabdomyosarcoma [43], and non-lymphoblastic leukemia [44,45,46], have been found to have increased incidence after prenatal in utero exposure, providing clinical evidence of inheritable mutagenicity.

Protein kinase B [47], AMP-activated protein kinase [48], Ca2+/CaM-dependent protein kinase β-kinase [49], mammalian target of rapamycin [50], pyruvate dehydrogenase kinase [51], hypoxia-inducible factor 1 [52], and peroxisome proliferator-activated receptor-γ [53] are just some of the cancer-related signaling pathways that cannabis has been shown to modulate.

Studies on the effects of cannabinoid-based drugs on immunity have shown that these drugs suppress a variety of cellular and cytokine mechanisms, particularly four: induction of apoptosis [54,55,56,57,58] (of T cells, macrophages, splenocytes, and thymocytes), inhibition of cell proliferation [59,60,61], inhibition of chemokine and cytokine production [62,63], and induction of Tregs [64].

Cannabinoids have been proven to be useful in the treatment of diseases such as gastric cancer [65], colorectal cancer [66], leukemia [67], and Sézary syndrome [68], according to some research. Moreover, the majority of cannabis and cannabinoid use in cancer patients has been for palliative care [69].

According to one study, three out of four patients want to find out information about cannabis from their cancer team, but only 15% receive it [70]. Only 30% of oncologists in the United States believe they are adequately trained to make informed cannabis recommendations [71], and 85% of oncology doctors in Minnesota would like more training on this topic [72].

Unfortunately, the lay press, especially on the Internet and social media, is full of claims about the “healing” effects of cannabis (fresh buds, dried cannabis, or “oil” products). These articles (usually written anonymously) extrapolate preclinical findings (using cell cultures or animal models) to humans without having any basis in fact [73].

The most recent literature review, which included both adult and pediatric patients, found 77 individual case reports describing patients with various cancers (breast, central nervous system, gynecology, leukemia, lung, prostate, and pancreas) who used cannabis as a treatment [74]. The data supporting 81% of these cases were considered to be weak. The investigators have established an online, anonymous survey of patients using cannabis for its anticancer effect to assess the impact of the botanical on malignancies (www.catasurvey.com, accessed on 22 June 2022) [75].

This comprehensive systematic review examines the effectiveness of cannabis and cannabinoids in cancer patients during their oncology therapies, as well as the major ongoing trials using cannabis in various cancer patients and specialties on ClinicalTrials.gov. The purpose of this review is to build on the current state of research on the topic of medical cannabis in cancer patients during their treatment and to explain the future promise it may offer. To our knowledge, no previous review has looked into the link between cannabis and cancer treatments in cancer patients and presented ongoing real-world clinical trials.

## 2. Materials and Methods

In 2006, the first study investigating the effectiveness of cannabis against cancer was published. A search using MEDLINE/PubMed database was performed between 1 January 2006 and 1 March 2022. Search terms included the following: cannabidiol, cannabis, CBD, dronabinol, endocannabinoids, medical marijuana, nabiximols, nabilone, THC, and cancer.

### 2.1. Data Collection Process

A total of 376 articles were found after using the above key terms as well as publication date and English filters in all databases. Finally, only five articles that met the inclusion criteria were included in the systematic review.

**Figure 1:**
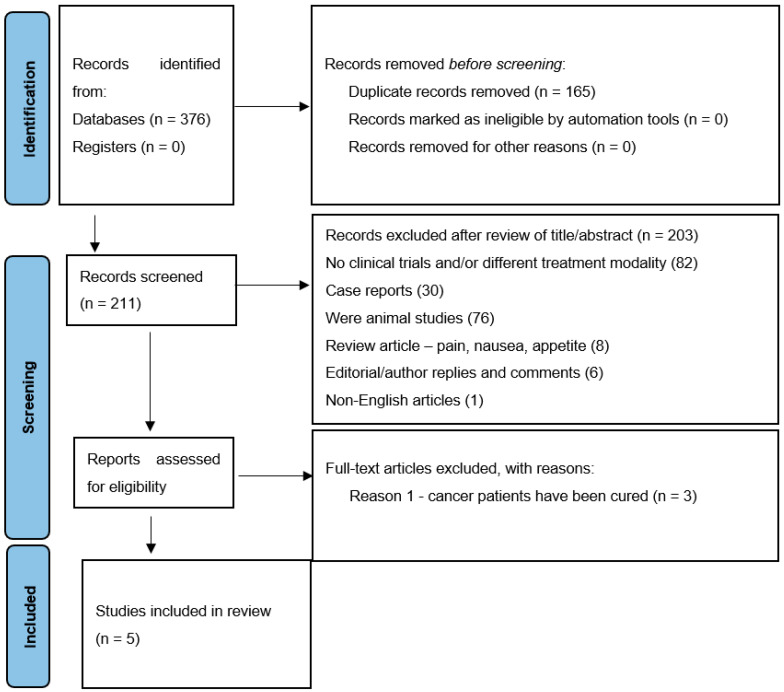
PRISMA flow diagram outlining the process of articles selected to be included in the review.

Figure 1 shows the selection process using the PRISMA 2020 flow diagram, where the data were methodically extracted.

### 2.2. Eligibility Criteria

Inclusion criteria for this review were (1) population: adults aged 18 and over diagnosed with all types of cancer; (2) context: oncological setting; (3) original research; (4) phenomena of interest: the effect of cannabis on anticancer treatment response was investigated; (5) published in English from the year 2006 forward with available full texts; (6) captured all types of medical cannabis and oncological drugs.

Articles were excluded if they did not contain original data, were not available in full text, were not in English, were not cancer-specific, or did not contain medical cannabis as described above, or if they were preclinical studies, review articles, systematic reviews, unpublished articles, dissertations, commentaries, meeting and conference proceedings, case reports, book reviews, opinion articles, or editorials.

A total of five articles were identified. Abstracts were reviewed and relevancy was determined for each of them (i.e., published in the English language and evaluated efficacy and safety of medical cannabis and cannabinoids in humans with cancer during their oncological treatments).

## 3. Results

According to the search strategy, 376 articles were found. However, 165 records were duplicated. After a review of titles and abstracts, 203 articles were excluded, including no clinical trials and/or different treatment modalities (82), case reports (30), animal studies (76), review articles (8), editorial/author responses and comments (6), and non-English articles (1).

### 3.1. Study Characteristics

The general characteristics of the studies listed in the review (*n* = 5) can be found in Table 1. Articles were published from 2006 to 2022 in a variety of scientific journals with different aims and scopes: British Journal of Cancer, Cancers, The Oncologist, and Frontiers in Oncology. All the records were written in English.

### 3.2. Design of the Studies

The first study was a randomized, placebo-controlled phase 1b clinical trial [76], the second was a prospective observational study [77], the third was a retrospective observational study [78], the fourth was a controlled pilot phase I clinical trial [79], and the last was a phase 2 randomized, double-blind clinical trial [80].

### 3.3. Participants and Regrouping

A total of 360 patients with various cancer localizations, while being under oncologic treatment, were enrolled in these studies. The number of participants in the studies ranged from 9 to 140 cancer patients. The first study [76] included 21 patients (12 in the active arm, 9 in the placebo arm) aged ≥18 years, with a histopathologically confirmed diagnosis of glioblastoma and evidence of initial disease progression after radiotherapy and first-line temozolomide (TMZ) chemotherapy. Patients had a status of ≥60% on the Karnofsky Performance Scale, and if they were taking steroids, it was a stable or reduced dose. Patients received nabiximols or placebo, with a maximum of 12 sprays/day with dose-intense temozolomide for up to 12 months. All patients received standard treatment (i.e., 6 weeks of radiotherapy with concomitant TMZ followed by adjuvant TMZ). The study protocol was registered in the United Kingdom (UK) on the Clinical-Trials.gov website (part 1: NCT01812603; part 2: NCT01812616). The second study [77] included 102 patients from the Division of Oncology at Rambam Health Care Campus in Haifa, Israel, who had metastatic cancer (stage IV) and had started checkpoint inhibitor therapy: 34 patients were taking cannabis (cannabis immunotherapy group: CI-G), while 68 were not (immunotherapy group: I-G). Approximately 70% of patients were men, and more than 50% had non-small cell lung cancer (NSCLC). The third study [78] included 140 patients from the Division of Oncology at Rambam Health Care Campus in Haifa, Israel, who were treated with nivolumab (89 nivolumab alone, 51 nivolumab plus cannabis) in 2015–2016 for advanced melanoma, non-small cell lung cancer, and clear cell renal cell carcinoma. The groups were similar in demographics and disease characteristics. The fourth study [79] was conducted in Madrid and included nine patients with glioblastoma, who had failed standard therapy surgery and external beam radiotherapy (60 Gy), in whom sequential magnetic resonance imaging showed clear evidence of tumor progression and who had a Karnofsky Performance Score of at least 60 (i.e., ability to function independently). The patients had received adjuvant tetrahydrocannabinol, which was administered intracranially. The fifth study [80] was conducted in Australia and enrolled 88 participants with a recurrent or inoperable high-grade glioma. Participants received oncological treatment and oil-based whole-plant cannabis extracts with a tetrahidrocanabinol:cannabidiol ratio of either 1:1 or 4:1.

## 4. Discussion

Cannabis products are only now beginning to be integrated into oncology clinical care. We have made great strides in our understanding of the physiology and pharmacology of the cannabinoid system in recent years. Although still strict, the legal situation regarding the use of cannabis-based medicines has improved, especially in response to the promising results of relevant basic research.

Cannabinoids have anticancer activity in cell lines and animal models, but well-designed human studies investigating their efficacy and safety are still lacking. In addition, the anticancer properties of cannabis must be balanced against their immunosuppressive properties, which may have pro-tumorigenic effects. These studies are continuing or have recently been completed, but the results have yet to be published.

Glioblastomas are the most common and aggressive form of brain tumors, with symptoms that are difficult to control [80]. These tumors can comprise more than 90% of the brain volume [81]. Almost all glioblastomas recur even after intensive treatment with surgery, radiotherapy, and chemotherapy, and the average survival time from diagnosis is only 12–18 months. Although Twelves et al. [76] found increased efficacy as measured by survival in patients treated with adjuvant nabiximols, any conclusions on efficacy are limited by small sample size and potentially confounding factors that differ between cohorts. According to the pharmacokinetic results, nabiximols had no significant effect on systemic TMZ exposure when administered as part of a dose-intensive temozolomide regimen.

With a better understanding of the effects of cannabinoid-based treatments on the immune system, we will be able to use them appropriately in combination with existing therapies to treat cancer patients. Immunotherapy has revolutionized cancer treatment in recent years by recovering tumor-induced immune deficit in the tumor microenvironment and modifying immune responses to a wide range of malignancies. Regarding the general effects on cannabis use in cancer patients, only one study has examined the interaction between cannabinoids and immunotherapy with checkpoint inhibitors. Bar-Sela et al. [77] suggest that exposure to cannabis before or during immunotherapy with immune checkpoint inhibitors may be associated with worsening success rates. Indeed, their data suggest that cannabis users are associated with shorter time to tumor progression (TTP) and shorter overall survival (OS). In addition, lymphocyte counts at baseline were lower in the cannabis user group, with higher counts positively correlated with treatment success rate. In this study, cannabis reduced some of the side effects of immunotherapy, such as skin toxicity, colitis, and thyroid disorders. However, a better understanding of the direct antitumor effect of cannabinoids and their influence on the immune system is essential for the integration of cannabinoids into the clinician’s armamentarium.

Taha et al. [78] found a possible interaction between cannabis use and immunotherapy in cancer patients with advanced malignancies, as evidenced by a decrease in the response rate to immunotherapy with cannabis use. Their analysis found no significant difference in OS or progression-free survival due to cannabis use. The authors also assert that factors affecting OS or progression-free survival (smoking, brain metastases, and poor performance status) are known to have a significant impact on these endpoints, independent of cannabis use.

Guzman et al. [79] conducted not only the first clinical trial to evaluate the antitumor effects of cannabinoids, but also the first-in-human study in which a cannabinoid was administered intracranially. Although intratumoral administration allows for high local concentration of the drug at the site, local perfusion through a catheter placed at the tumor site is an obvious limitation of the technique in large tumors such as those treated in their study. The median survival time of the cohort from the start of cannabinoid administration was 24 weeks, suggesting a benefit.

An Australian study performed by Schloss et al. [80] on the tolerability of a single nightly dose of two cannabis oils in patients with high-grade gliomas receiving standard therapies was recently reported. Participants received treatment with oil-based whole-plant cannabis extracts with a THC:CBD ratio of either 1:1 or 4:1. Of the 83 participants who completed at least 4 weeks of the intervention, 90% had glioblastomas and 10% had anaplastic astrocytomas. Sixty-one patients completed the 12-week study. Physical and functional domains of quality of life and sleep were improved in the group with a THC:CBD ratio of 1:1 compared with the group with a ratio of 4:1. Although the primary objective was to assess tolerability of the two ratios, MRI scans were performed in 53 participants at baseline and after 12 weeks because disease status was a secondary outcome. After 12 weeks, disease had regressed in 11%, was stable in 34%, had T2 flair and mild enhancement in 16%, and had progressed in 10%. No differences in treatment outcomes were observed between the groups.

Given the lack of clinical evidence on the side effects and potential risks of cannabis use during cancer treatment in patients, oncologists should also carefully consider the potential benefits of medical cannabis before prescribing it. A recent meta-analysis showed no beneficial effect of nabiximols for cancer pain [82].

Possible risks of cannabis use include increased anxiety [83] and panic attacks [84], exacerbation of existing mood disorders or psychosis [84], impairment of cognitive function and increased risk of traffic accidents [84], and addiction [85]. THC is considered to trigger psychosis [86], but another component, CBD, seems to work against it [87]. Medical cannabis may relieve anxiety, nausea, neuropathy, vomiting, and appetite and weight loss [88].

*Where are we going?*

Despite advances in cancer diagnosis and treatment, the problem of cancer metastasis remains unsolved [89]. Approximately 90% of cancer patients die due to progression of metastatic disease. Targeting the lethal targets after finding the tumor-specific mutations is a viable strategy for cancer treatment. To our knowledge, there are several knowledge gaps that would benefit from additional clinical trials researching the impact of cannabis on cancer treatment, including the impact of cannabis on the treatment of oncogenic mutations (e.g., HRAS, KRAS, NRAS, BRAF, and EGFR). It would also be helpful to measure and analyze the immunological landscape of cannabis treatment using a panel of T cells, B cells, neutrophils, and macrophages for immunohistochemistry during targeted therapy. In-depth explorations of different subtypes of cancers, such as breast, colorectal, and lung cancer in advanced and metastatic stages, and cannabis use in combination with standard or experimental anticancer therapies, with different cannabinoid types/doses, and with oral or inhaled routes of administration would also be important research avenues.

Medical centers around the world are trying to assess the efficacy of approved cannabis given to people with different types of malignancies and to evaluate potential factors that influence cancer pain, nausea, and anticancer efficacy. As of June 2022, ClinicalTrials.gov [90] listed 23 ongoing studies of the efficacy of cannabis in the cancer population. Only one study addressed the efficacy of cannabis in combination with oncologic treatment. This list includes both intervention and observational studies, as well as cohort studies, with an estimated number of participants of up to 218,000 and an estimated study completion date of December 2022.

## 5. Conclusions

Cannabis use is expected to increase in more states following its legalization. Therefore, it is important to accelerate knowledge about cannabis use in this vulnerable patient group. In summary, cannabinoid-based treatments have beneficial palliative properties in cancer patients and may have antitumor effects in certain cancer subtypes.

These findings highlight the complexity of the use of cannabinoid-based medicines and the need for further comparative scientific research. The interactions of cannabinoids with conventional cytotoxic agents need to be clearly defined. These findings have led us to conclude that further extensive research is needed to confirm the possibility of using cannabis in cancer treatment.

Furthermore, health professionals can play an active role in the treatment of patients by identifying patients who may benefit from cannabis and cannabinoids, monitoring and educating patients who use these products, and participating in cannabis and cannabinoid research and education for health professionals.

### Limitations

Evidence gaps remain for most cancers studied. In the area of well-structured clinical trials, cancer care provides an opportunity to conduct controlled trials to further investigate the potential benefits of cannabis to improve cancer care. However, the identification of several registered randomized controlled trials nearing completion suggests that better evidence will be available in the coming years.

More long-term research is needed to determine the long-term consequences of cannabis use on cancer treatment and the tumor itself. There are few data to evaluate the complexity of cancer treatment in terms of the effectiveness of cannabis during or after cancer treatment. We also do not know whether the dynamic metabolic and immunologic effects of chemotherapy and cancer treatment may alter the pharmacodynamics or pharmacokinetic properties of cannabis or its derivatives.

## Figures and Tables

**Table 1:** Data extraction table in order of medical cannabis intervention type and date, highlighting author, country, study type, number of participants, intervention, administration, daily dose, dosing schedule, duration, outcome measures, primary outcomes, strengths and limitations, and link for articles included in the review.

Author	Country	Study Type	Number	Intervention	Administration	Daily Dose	Dosing Schedule	Duration	Outcome Measures	Primary Outcomes	Strengths and Limitations	Link
Twelves et al., 2021 [76]	United Kingdom [UK]	Phase 1b randomized, double-blind, placebo-controlled clinical trial	21 (12 in the active arm, 9 in placebo)	Sativex® (Nabiximols spray)	Oromucosal spray	Up to 12 sprays or 30 mg CBD/32.4 mg THC	Controlled	24.9 weeks for the Sativex ® group and 23.6 weeks for the placebo group	Magnetic resonance (MRI)	Co-administration of the Sativex^®^ in cancer patients treated with temozolomide demonstrated that, in the Sativex^®^ group, the overall survival benefit was 21.8 months compared with 12.1 months for the placebo group. One-year survival in favor of nabiximols was statistically significant (*p* = 0.042).	Strengths − individualized titration and personalized dosing of nabiximols − without randomization and placebo control, interpretation of the OS in patients treated with nabiximols would have been confounded Limitations − the small number of patients	https://www.nature.com/articles/s41416-021-01259-3(accessed on 5 January 2022)
Bar-Sela et al., 2020 [77]	Israel	Prospective observatory study	102 (68 immunotherapy and 34 immunotherapy (anti-PD-1 (Pembrolizumab or Nivolumab; IPIlimumab and Nivolumab) and anti-PD-L1 (Durvalumab or Atezolizumab)) plus cannabis)	Cannabis oil, combined oil and flowers	The use of cannabis had been started nine months to two weeks before the first immunotherapy treatment. The patients had permission to change cannabis products monthly.	Up to 40 g per month of cannabis	Uncontrolled	11–14 weeks of treatment	Panel of serum endocannabinoids (eCBs) and eCB-like lipids	Initiating immunotherapy with cannabis use negatively affects OS and time to tumor progression of cancer patients treated with immunotherapy. The median survival was 6.4 months in those using cannabis and 28.5 months in those who were not. The patient group using cannabis (34 patients) were found to have a statistically significant reduction in the rate of response to immunotherapy agents and also a significantly shorter time to progression (*p* = 0.0025) and reduced overall survival (*p* = 0.00094) when compared to the group of non-cannabis users (68 patients). The cannabis user group also experienced fewer treatment-related adverse events when compared to the non-using patients (*p* = 0.057).	Strengths − the first study evaluating the impact of cannabis use during immunotherapy treatment − the first “red flag” for using cannabis as a palliative treatment in advanced cancer patients starting immunotherapy—and suggests that its use should be carefully examined Limitations − a relatively small group of patients in the main clinical categories, such as different cancer types and diverse lines of oncology treatment − specific characteristics of the tumor, the patient, or the type of immunotherapy treatment may have influences that were not evaluated well due to the sample size	https://www.mdpi.com/2072-6694/12/9/2447(accessed on 5 January 2022)
Taha et al., 2019 [78]	Israel	Retrospective observational study	140 patients (89 nivolumab alone, 51 nivolumab plus cannabis) with stage IV non-small cell lung cancer (NSCLC) or clear cell renal cell carcinoma (RCC) or advanced melanoma	Cannabidiol, tetrahydrocannabinol	Smoked or inhaled (cannabis flowers only), prepared cannabis oil, or combined use	Up to 30 g per month of cannabis	Uncontrolled	1 year	The response rate was evaluated using RECIST criteria based on imaging assessments carried out every 11–14 weeks.	Cannabis users showed a less favorable prognosis in terms of response rate (RR), which was reduced in the nivolumab–cannabis group compared to the nivolumab group (*p* = 0.016). Cannabis use did not significantly influence the progression-free survival (PFS) or the overall survival (OS). Cannabis composition had no influence on the results.	Strengths − given the high number of patients diagnosed with NSCLC, a comparison was made between them and the other malignancies (melanoma and clear cell RCC) − specific attention was paid to the use of concomitant antibiotics or glucocorticosteroids Limitations − given the high number of lung cancer patients, the study included a limited number of patients and a nonrepresentative sample − the follow-up period was relatively short − retrospective data with a nonrepresentative sample	https://pubmed.ncbi.nlm.nih.gov/30670598/(accessed on 5 January 2022)
Guzmán et al., 2006 [79]	Spain	Pilot phase I controlled clinical trial	9 patients with glioblastoma	Δ9-Tetrahydrocannabinol (THC)	Intratumorally	Daily intracranial administration of delta-9 THC	Total doses ranging from 0.8 mg to 3.29 mg	Range of 10–64 days	Biopsies of the treated tumors, MRI	Δ9-Tetrahydrocannabinol inhibited tumor-cell proliferation in vitro and decreased tumor-cell Ki67 immunostaining when administered to two patients. Median survival rate from the surgical operation of tumor relapse was 24 weeks. Two of the patients (3 and 8) survived for approximately 1 year.	Strengths − this is the first human study in which a cannabinoid is administered intracranially in patients with recurrent glioblastoma − ↓ tumor cell Ki67 Limitations − a relatively small group of patients − invasive, traumatic route of THC administration − owing to the characteristics of this study, the effect of THC on patient survival was unclear, and an evaluation of survival rate would require a larger trial with a different design	https://www.ncbi.nlm.nih.gov/pmc/articles/PMC2360617/.(accessed on 5 January 2022)
Schloss et al., 2021 [80]	Australia	Phase 2 randomized, double-blind clinical trial	88 patients with high-grade glioma	THC and cannabidiol (CBD)	Oil ingested orally	1:1 THC 4.6 mg/mL: cannabidiol (CBD) 4.8 mg/mL and 4:1 THC 15 mg/mL: CBD 3.8 mg/mL	Controlled	12 weeks	The Functional Assessment of Cancer Therapy—Brain (FACT-Br), participant diary and MRI results imaging assessments	Physical and functional domains of quality of life and sleep were improved in the group with a THC:CBD ratio of 1:1 compared with the group with a ratio of 4:1. Although the primary objective was to assess tolerability of the two ratios, MRI scans were performed in 53 participants at baseline and after 12 weeks because disease status was a secondary outcome. After 12 weeks, disease had regressed in 11%, was stable in 34%, had T2 flair and mild enhancement in 16%, and had progressed in 10%. No differences in treatment outcomes were observed between groups.	Strengths − no published study was found that was similar to this study in terms of oral administration of the intervention, outcome measurement, dosage of the intervention, or time frame Limitations − there was no placebo group, which is considered the gold standard in randomized clinical trials − the differences between the treatments in the retrospective cases and the study population also limits the comparison between these groups	https://www.frontiersin.org/articles/10.3389/fonc.2021.649555/full(accessed on 5 January 2022)
